# Supplementary material for: Circuit analysis of the Drosophila brain using connectivity-based neuronal classification reveals organization of key communication pathways
Source: Netw Neurosci. 2023 Jan 1;7(1):269–98. doi: 10.1162/netn_a_00283 (PMC10275213; doi:10.1162/netn_a_00283)
Supplement: Supplementary file 5 [file netn-7-1-269-s005.pdf]

Mehta, K., Goldin, R. F., & Ascoli, G. A. (2023). Supporting information for "Circuit analysis of the Drosophila brain using connectivity-based neuronal classification reveals organization of key communication pathways. *Network Neuroscience, 7*(1), 269–298. https://doi.org/10.1162/netn_a_00283

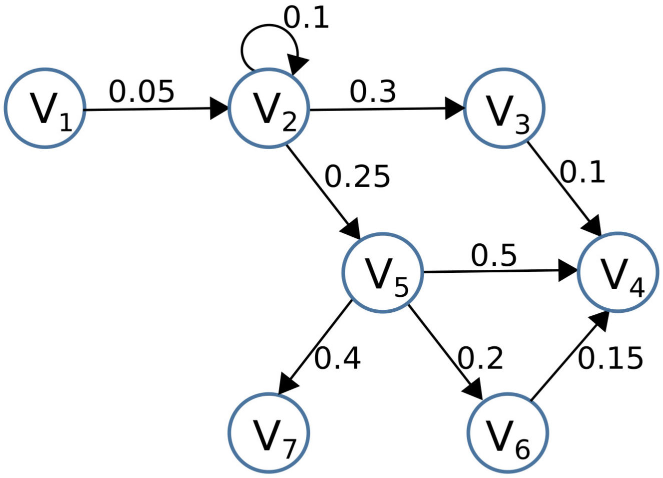

Number of neurons in *i*-th class

i	$\lvert V_i \rvert$	$\lvert \tilde{V}_i \rvert$
1	1000	10
2	250	2.5
3	100	1
4	500	5
5	300	3
6	400	4
7	150	1.5

Block connection prob. p_{ij}

0	0.05	0	0	0	0	0
0	0.1	0.3	0	0.25	0	0
0	0	0	0.1	0	0	0
0	0	0	0	0	0	0
0	0	0	0.5	0	0.2	0.4
0	0	0	0.15	0	0	0
0	0	0	0	0	0	0

Cost $c_{ij} := \dfrac{1}{p_{ij}|\widetilde{V}_i||\widetilde{V}_j|}$

0	0.8	0	0	0	0	0
0	0	1.33	0	0.53	0	0
0	0	0	2	0	0	0
0	0	0	0	0	0	0
0	0	0	0.13	0	0.42	0.56
0	0	0	0.33	0	0	0
0	0	0	0	0	0	0

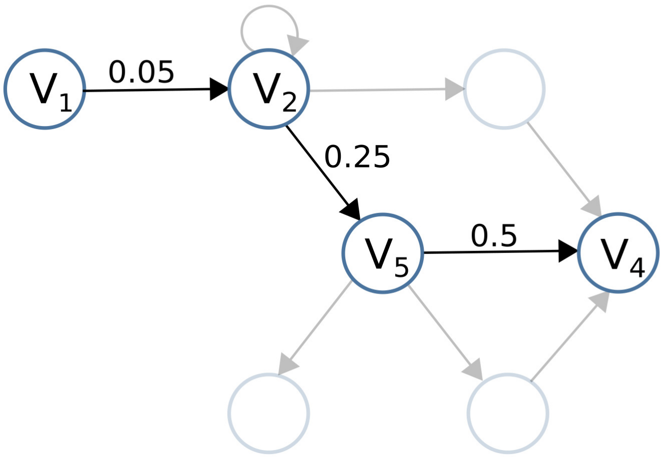

Path:

$$\mathcal{P} = \{V_1, V_2, V_5, V_4\}$$

Path length:

$$\ell(\mathcal{P}) = c_{12} + c_{25} + c_{54}$$

$$= 0.8 + 0.53 + 0.42$$

$$= 1.75$$

Figure S1: A simple example illustrating how the path length of a random walk is calculated, using a mock circuit with seven classes (blocks). The number of neurons in each class $|V_i|$, and directional edge weights between classes p_{ij} are assumed to be known — which are used to calculate the cost c_{ij} of each random step in the walk. The path length for the random walk $V_1 \to V_2 \to V_5 \to V_4$ is then obtained by summing the costs along the traversed path.
