# Supplementary material for: Circuit analysis of the Drosophila brain using connectivity-based neuronal classification reveals organization of key communication pathways
Source: Netw Neurosci. 2023 Jan 1;7(1):269–98. doi: 10.1162/netn_a_00283 (PMC10275213; doi:10.1162/netn_a_00283)
Supplement: Supplementary file 6 [file netn-7-1-269-s006.pdf]

Mehta, K., Goldin, R. F., & Ascoli, G. A. (2023). Supporting information for "Circuit analysis of the Drosophila brain using connectivity-based neuronal classification reveals organization of key communication pathways. Network Neuroscience, 7(1), 269–298. https://doi.org/10.1162/netn_a_00283

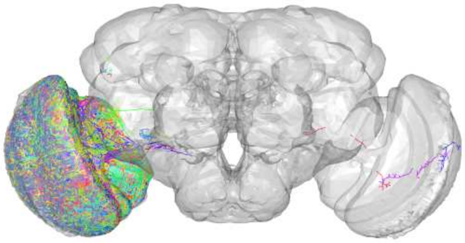

1

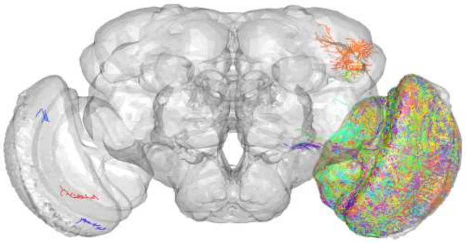

2

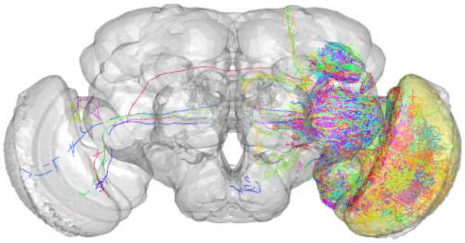

3

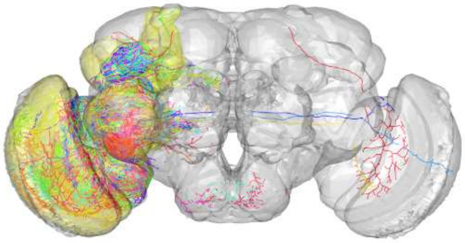

4

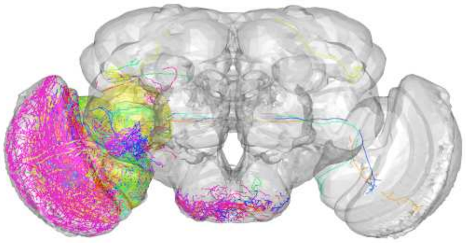

5

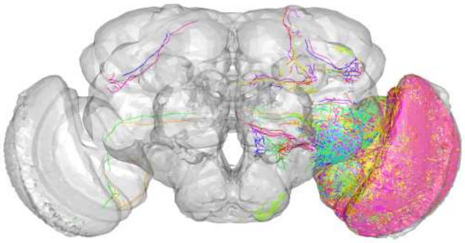

6

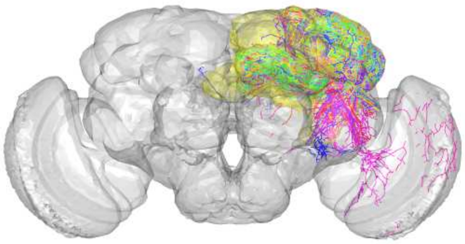

7

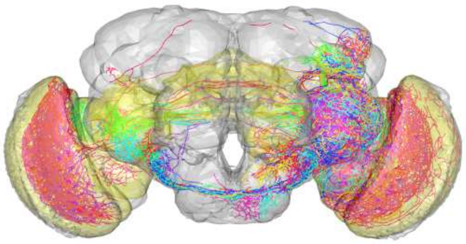

8

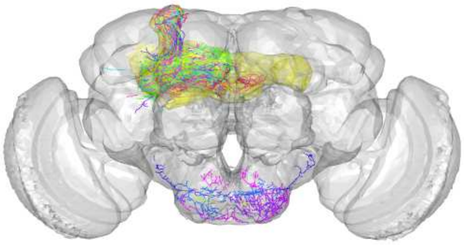

9

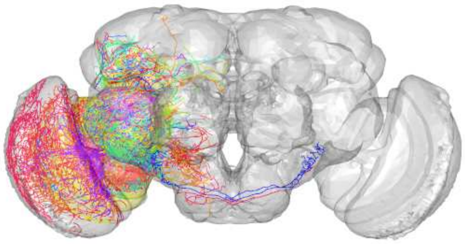

10

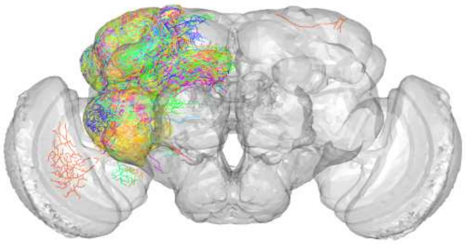

11

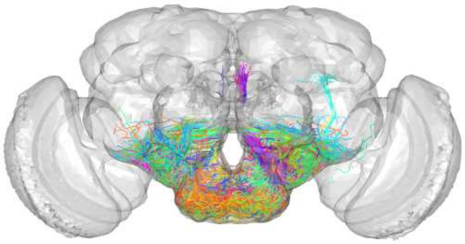

12

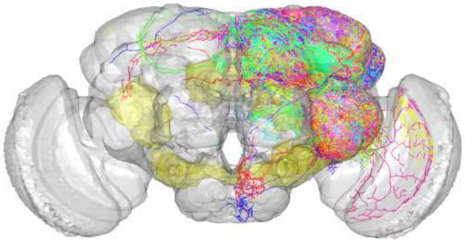
13

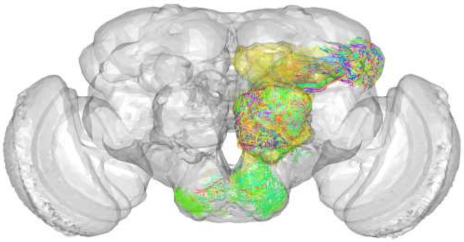
14

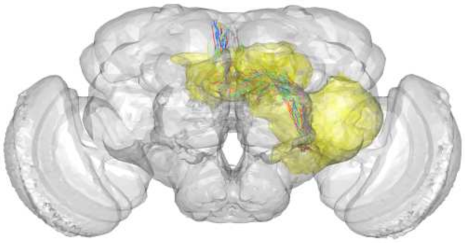
15

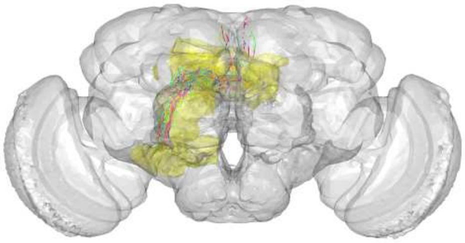
16

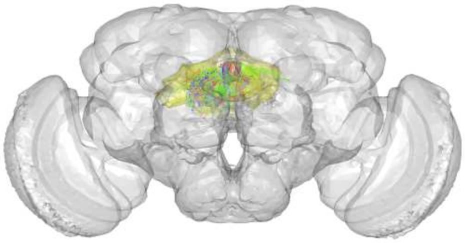
17

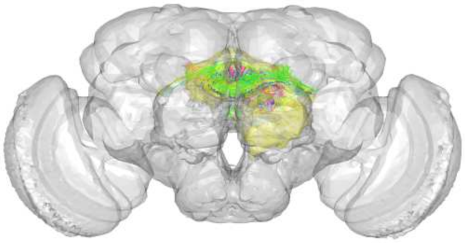
18

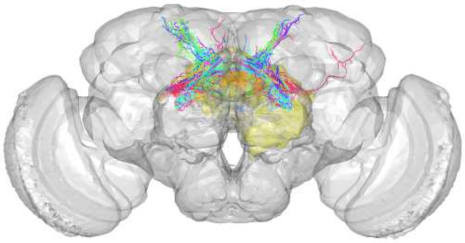
19

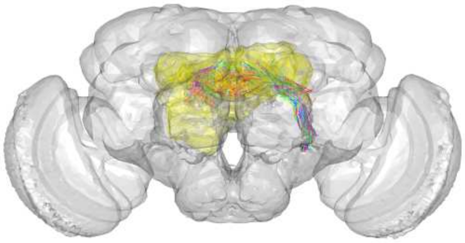
20

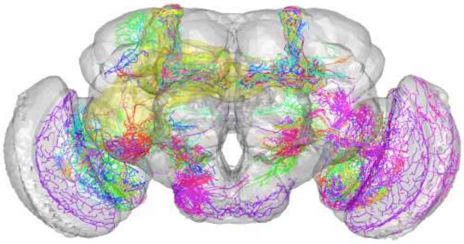
21

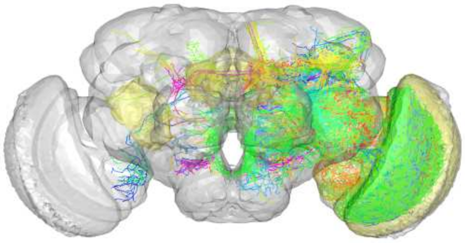
22

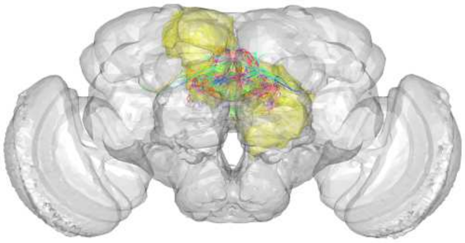
23

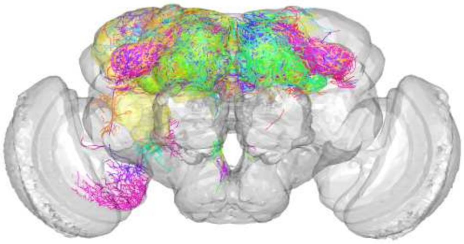
24

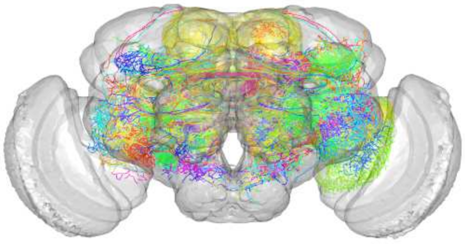

25

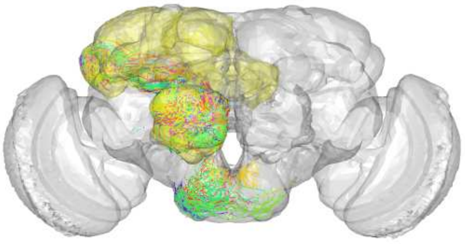

26

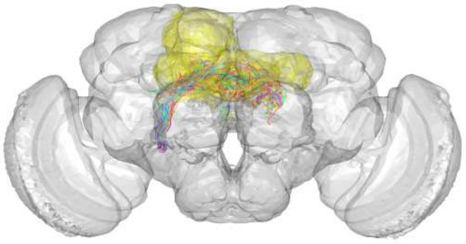

27

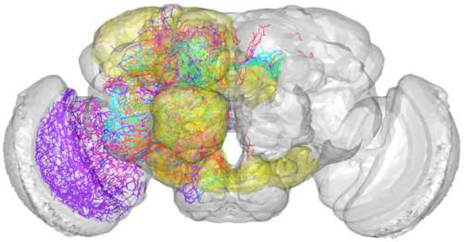

28

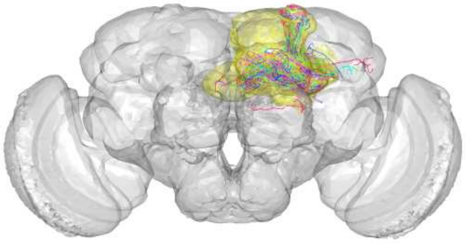

29

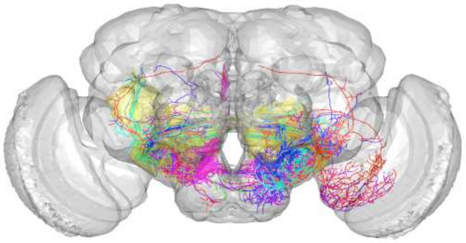

30

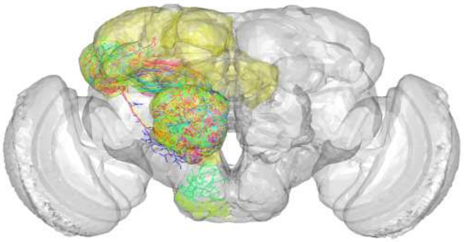

31

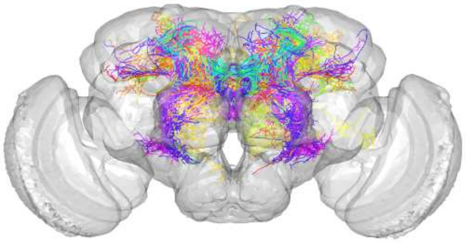

32

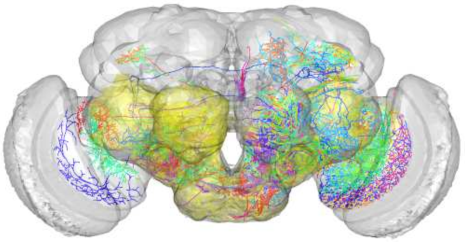

33

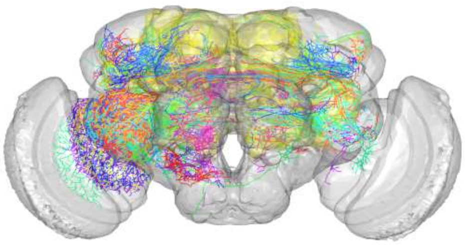

34

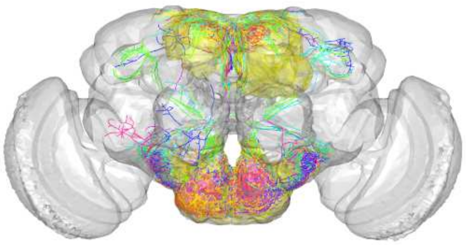

35

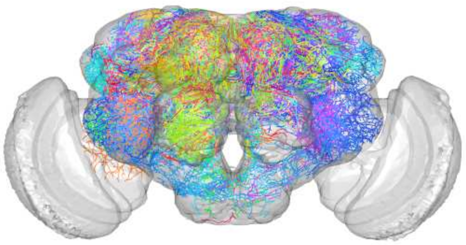

36

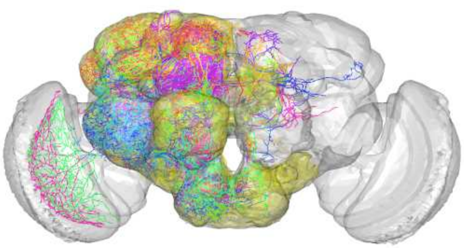

37

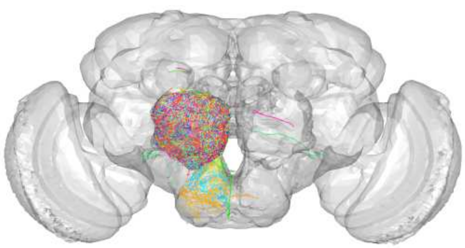

38

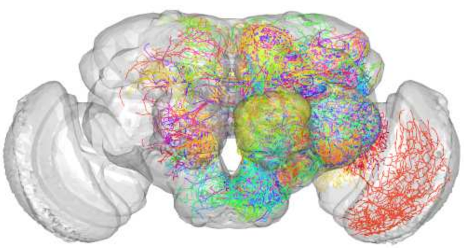

39

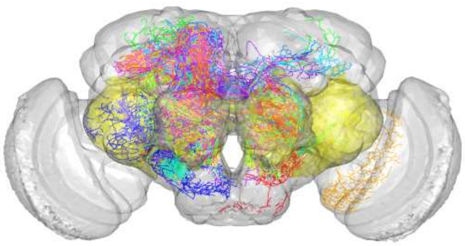

40

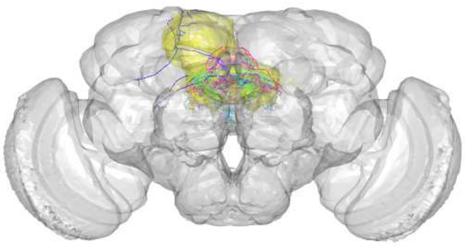

41

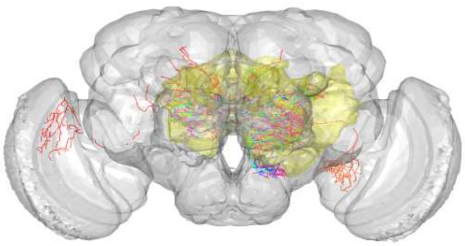

42

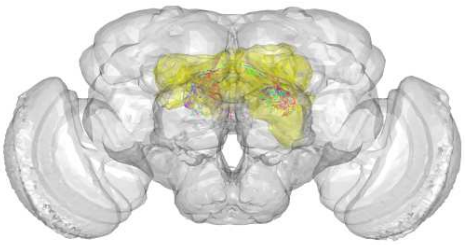

43

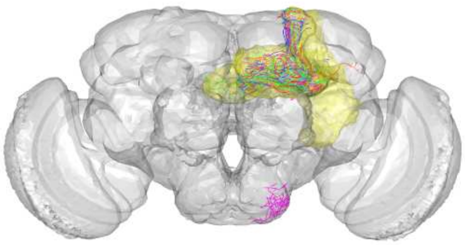

44

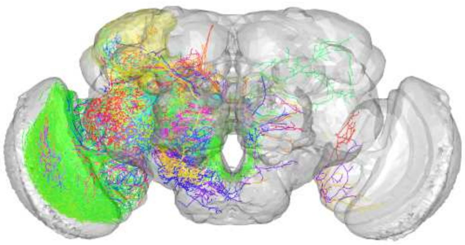

45

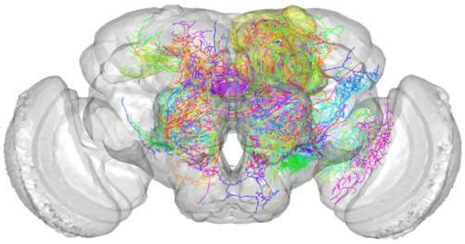

46

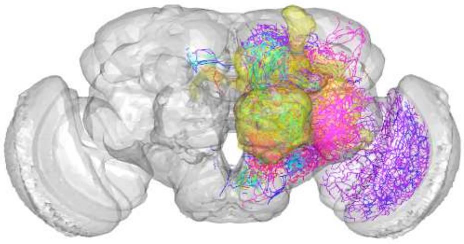

47

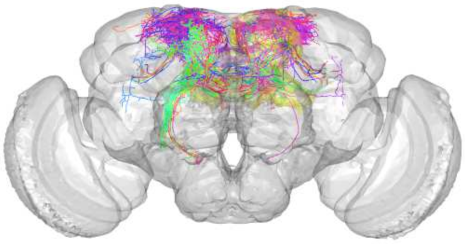

48

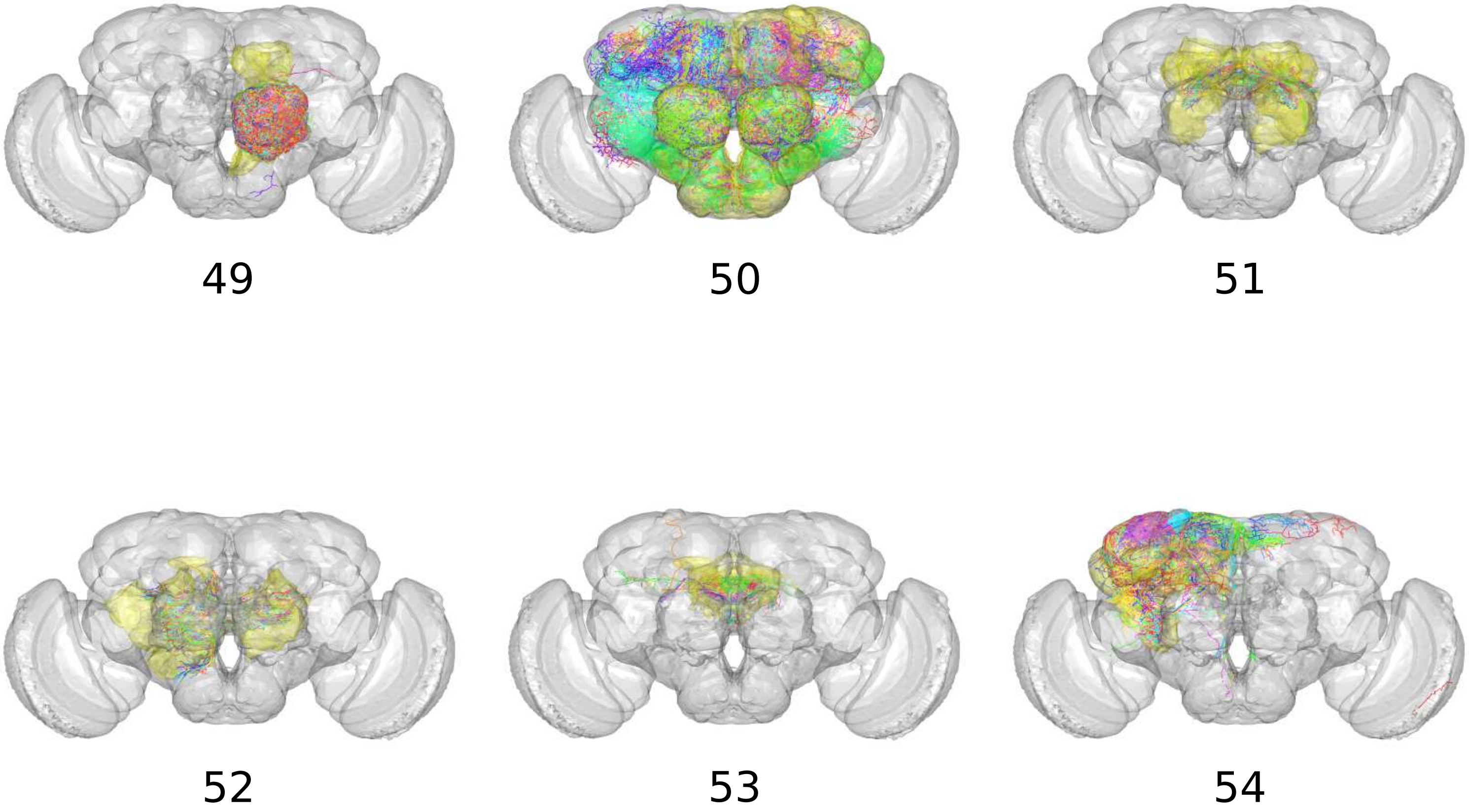

Figure S2: Each connectivity-based class embedded using the `natverse` 3D template of the Drosophila brain, showing its constituent neurons and innervating neuropils.
