# Supplementary material for: Circuit analysis of the Drosophila brain using connectivity-based neuronal classification reveals organization of key communication pathways
Source: Netw Neurosci. 2023 Jan 1;7(1):269–98. doi: 10.1162/netn_a_00283 (PMC10275213; doi:10.1162/netn_a_00283)
Supplement: Supplementary file 7 [file netn-7-1-269-s007.pdf]

Mehta, K., Goldin, R. F., & Ascoli, G. A. (2023). Supporting information for "Circuit analysis of the Drosophila brain using connectivity-based neuronal classification reveals organization of key communication pathways. *Network Neuroscience*, 7(1), 269–298. https://doi.org/10.1162/netn_a_00283

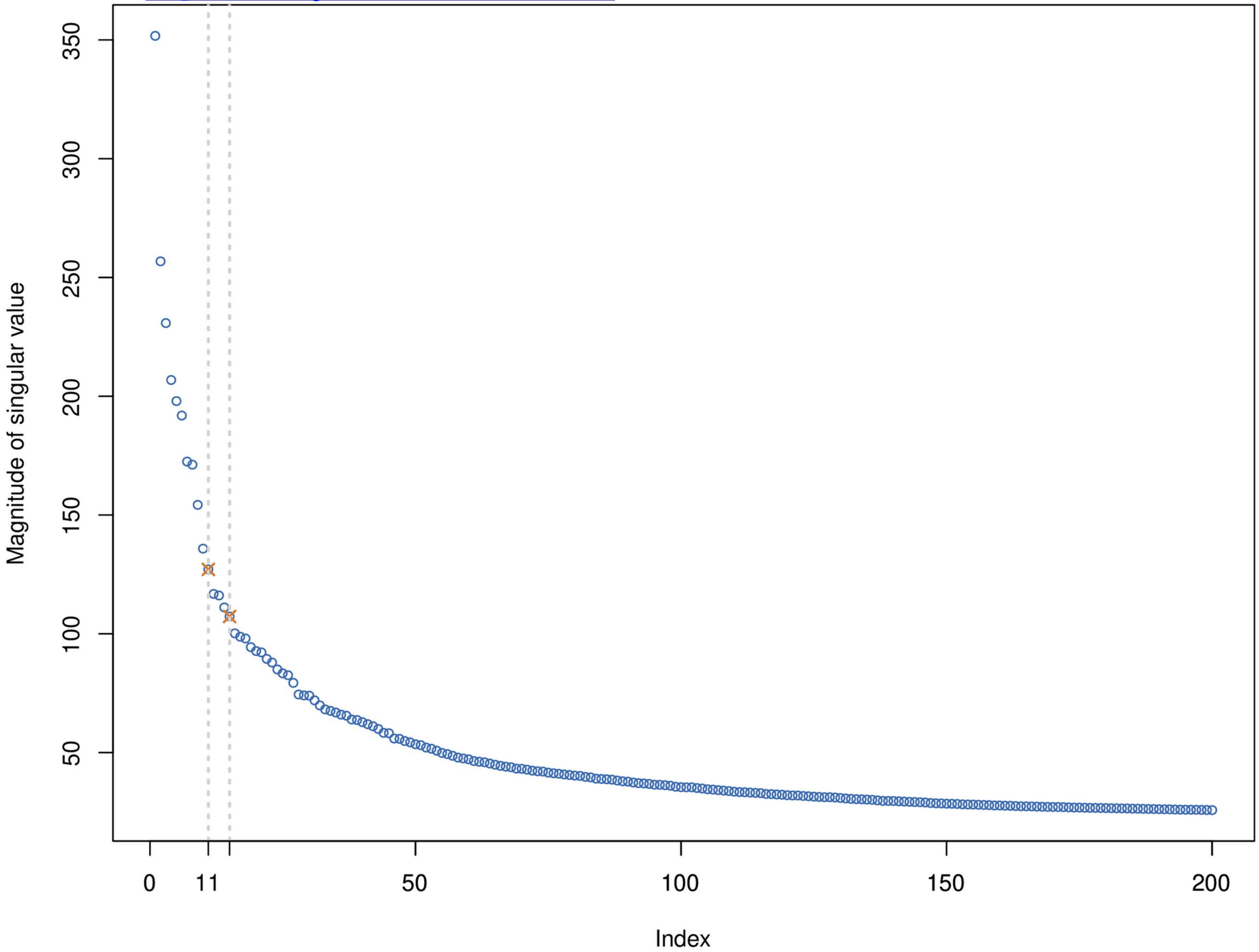

Figure S3: The possible choices for the embedding dimensionality *d={11,15}* were determined by identifying the first and second elbow-point (Zhu and Ghodsi, 2006), respectively, on the scree plot of singular values.
