# Supplementary material for: Circuit analysis of the Drosophila brain using connectivity-based neuronal classification reveals organization of key communication pathways
Source: Netw Neurosci. 2023 Jan 1;7(1):269–98. doi: 10.1162/netn_a_00283 (PMC10275213; doi:10.1162/netn_a_00283)
Supplement: Supplementary file 8 [file netn-7-1-269-s008.pdf]

Mehta, K., Goldin, R. F., & Ascoli, G. A. (2023). Supporting information for "Circuit analysis of the Drosophila brain using connectivity-based neuronal classification reveals organization of key communication pathways. *Network Neuroscience, 7*(1), 269–298. https://doi.org/10.1162/netn_a_00283

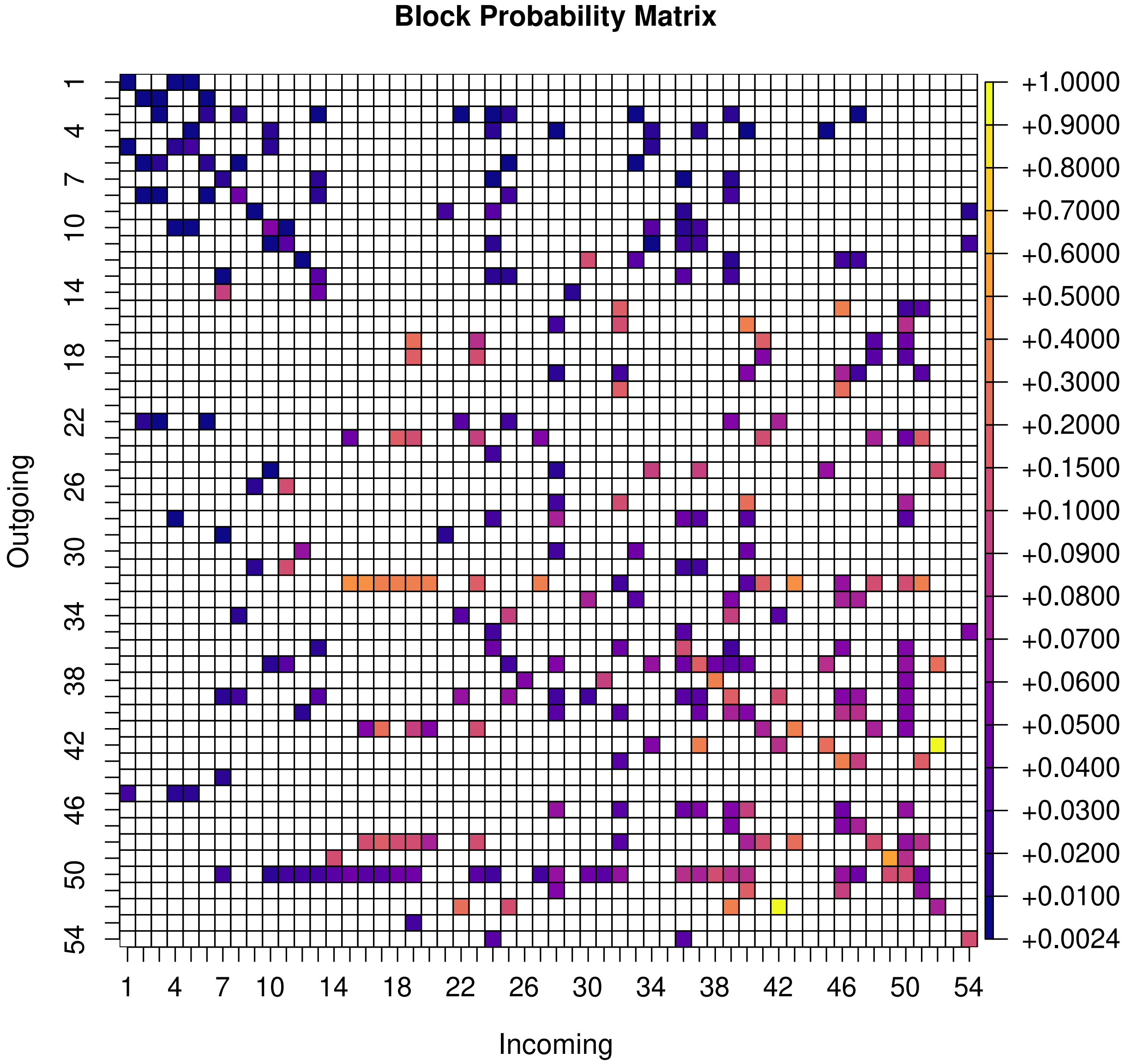

Figure S4: **(a)** The estimated block connectivity probabilities $\hat{p}_{ij}$'s between the 54 connectivity-based classes. The 319 non-zero $\hat{p}_{ij}$'s represent a weighted, directed edge between the 54 classes in the inferred mesoscale circuit.

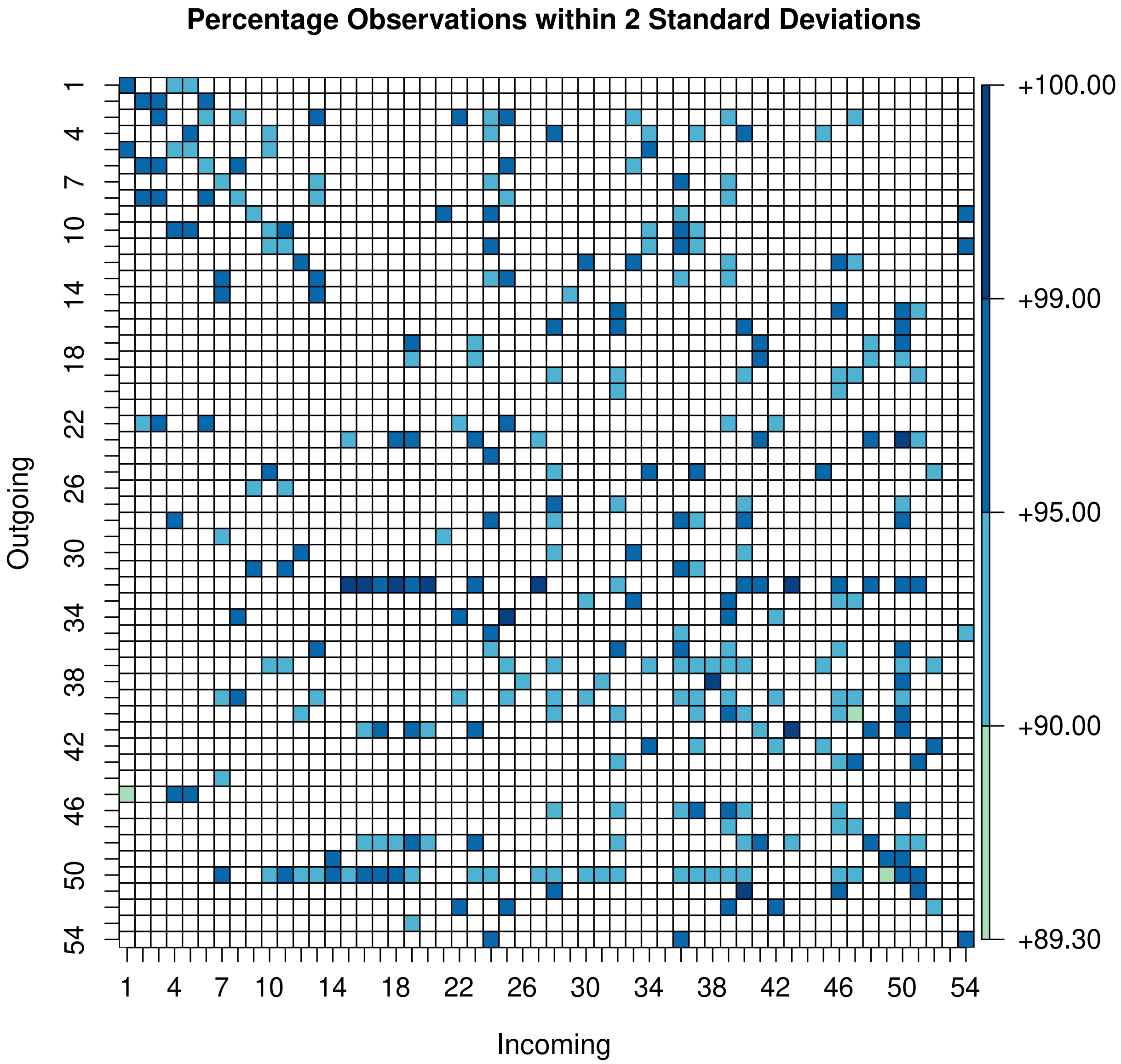

Figure S4: **(b)** The percentage of observed values for a block-pair that lie within two standard deviations of the expected binomial mean $|V_j|\hat{p}_{ij}$.

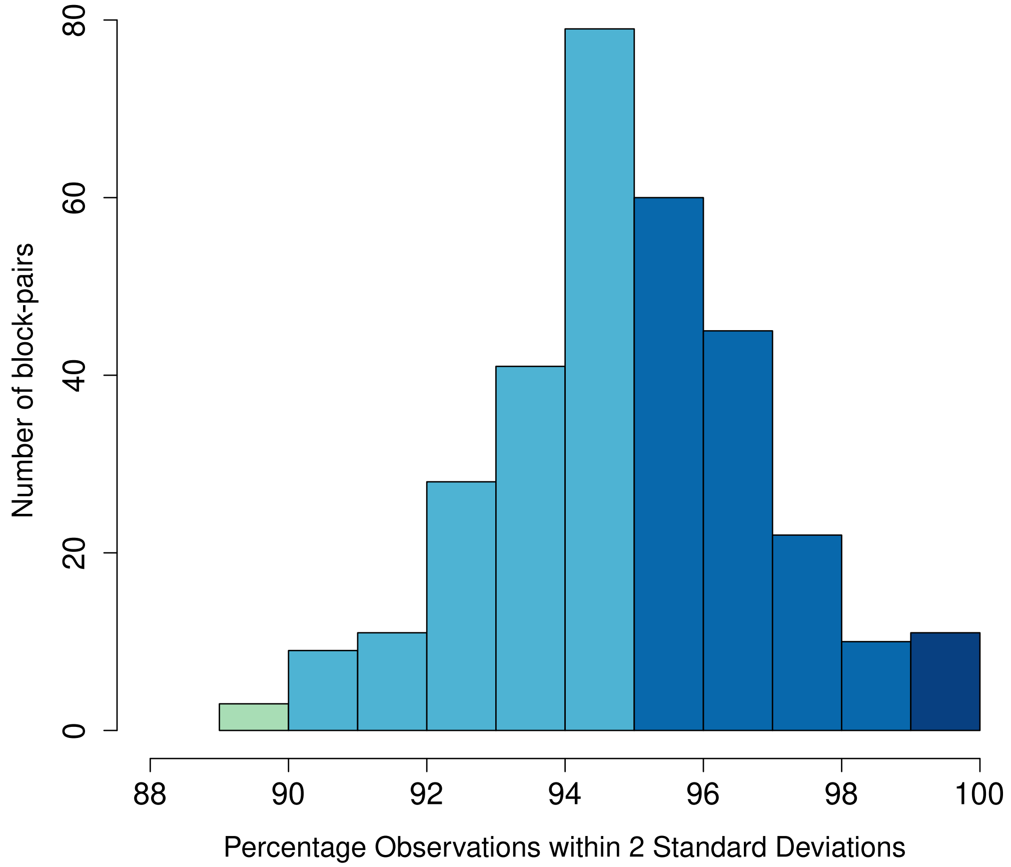

Figure S4: (c) A histogram showing the frequency count of block-pairs by the percentage of observed values that lie within two standard deviations of the expected binomial mean $|V_j|\hat{p}_{ij}$.
